# Oral Dysesthesia Rating Scale: a tool for assessing psychosomatic symptoms in oral regions

**DOI:** 10.1186/s12888-014-0359-8

**Published:** 2014-12-21

**Authors:** Akihito Uezato, Akira Toyofuku, Yojiro Umezaki, Motoko Watanabe, Akira Toriihara, Makoto Tomita, Naoki Yamamoto, Akeo Kurumaji, Toru Nishikawa

**Affiliations:** Department of Psychiatry and Behavioral Sciences, Tokyo Medical and Dental University Graduate School, 1-5-45 Yushima, Bunkyo-ku, Tokyo, 113-8510 Japan; Department of Psychiatry and Behavioral Neurobiology, University of Alabama at Birmingham, 1713 6th Avenue South, Birmingham, AL 35294 USA; Section of Psychosomatic Dentistry, Tokyo Medical and Dental University Graduate School, 1-5-45 Yushima, Bunkyo-ku, Tokyo, 113-8510 Japan; Department of Psychiatry, Medical University of South Carolina, 67 President Street, Charleston, SC 29425 USA; Department of Diagnostic Radiology and Oncology, Tokyo Dental and Medical University Graduate School, 1-5-45 Yushima, Bunkyo-ku, Tokyo, 113-8510 Japan; Clinical Research Center, Tokyo Medical and Dental University Hospital Faculty of Medicine, 1-5-45 Yushima, Bunkyo-ku, Tokyo, 113-8510 Japan

**Keywords:** Cenesthopathy, Somatic-type delusional disorder, Somatoform disorder, Delusional parasitosis, Validity, Reliability, Oral DRS

## Abstract

**Background:**

The concept of cenesthopathy was first introduced by Dupré and Camus in 1907 to describe clinically unexplainable bodily sensations mainly attributed to psychiatric pathology. If it occurs in oral regions, it is termed oral cenesthopathy and it has been of special interest to psychiatrists and dentists. While there is no independently defined criteria for this condition, which is classified as either a delusional or a somatoform disorder, clinical practice and research require a standard scale to measure and rate its symptoms. In this study, we included any types of psychosomatic symptoms in oral regions as oral dysesthesia, and developed an Oral Dysesthesia Rating Scale (Oral DRS) and evaluated its validity and reliability as an assessment tool.

**Methods:**

The scale was developed based on literature review and extensive clinical experience. Twelve reviewers assessed relevancy of each item to oral dysesthesia symptoms by 1–4 scoring scale and item content validity index was computed. To evaluate the inter-rater reliability of Oral DRS, pairs of raters administered the scale to 40 randomly selected patients with complaints of oral dysesthesia symptoms and Cohen’s weighted kappa coefficient was determined for each item.

**Results:**

The scale assesses the severity of feelings of foreign body [A1], exudation [A2], squeezing-pulling [A3], movement [A4], misalignment [A5], pain [A6], and spontaneous thermal sensation or tastes [A7], and the degree of impairment in eating [B1], articulation [B2], work [B3], and social activities [B4] on a scale of 0–5. Items A1, A2, A3, A4, B3, and B4 demonstrated acceptable content validity. Inter-rater reliabilities were good or excellent for all items evaluated.

**Conclusion:**

The Oral DRS can help define the nosography of clinically unexplainable oral dysesthesia through further case evaluation and clinical research and facilitate devising of treatment modalities.

**Electronic supplementary material:**

The online version of this article (doi:10.1186/s12888-014-0359-8) contains supplementary material, which is available to authorized users.

## Background

Clinically unexplainable somatic symptoms occurring in patients mainly suffering from psychotic disorders, which is termed “cenesthopathy,” was first described by Dupré and Camus in 1907 [1,2]. In the history of Japanese medicine, which has been largely influenced by French and German medicine, the concept of cenesthopathy was introduced by Hozaki [3]. He reported cases of patients complaining of chronic unusual sensations especially in oral regions and designated it as “oral cenesthopathy”. Its nosography has been discussed in case reports and reviews in contemporary Japanese psychiatry [4-12], but overlooked in mainstream medicine. According to the University Psychiatry Clinic in Japan [5], 18 out of 10,278 outpatient cases (0.175%) seen in 5.5 years were diagnosed as oral cenesthopathy. Patient complaints included unusual and uncomfortable sensations in the mouth such as over-secretion of mucus, tingling, burning, pain, pulling on the teeth, moving teeth, foreign bodies, and unusual tastes without a somatic base. While infrequent, this condition desires clinical attention because patients usually visit multiple physicians and dentists seeking treatment and often claim that the treatment itself worsens their symptoms.

The diagnosis of oral cenesthopathy is still controversial, and contemporary psychiatry does not provide independently defined diagnostic criteria. Hozaki suggested that cenesthopathy occurring alone should be differentiated from that showing comorbidity with other psychotic, mood, or organic brain disorders [13]. In our clinical experience, abnormal somatic sensations that are unexplainable and delusional tend to be considered classical cenesthopathy. For example, if a patient complains “a wire comes out of my gum and coils around my teeth”, this condition is categorized as a delusional disorder, somatic type (297.1) in the Diagnostic and Statistical Manual of Mental Disorders, Fourth Edition (DSM-IV), and as a delusional disorder (F22.0) in the ICD-10 Classification of Mental and Behavioural Disorders (ICD-10). However, a complain like “something sticky is always in my mouth and annoys me” is not absurd but does indicate a pathological condition. A patient with such a complain will be categorized to have a somatoform disorder. Commonly described “burning mouth syndrome” is categorized as a pain disorder (307.8 in DSM-IV and F45.4 in ICD-10). Here in this paper, to include any types of psychosomatic symptoms in oral regions with psychotic or somatoform features as well as comorbidities with other mental illness, abnormal oral sensations are consistently referred to as “oral dysesthesia”.

Regardless of diagnostic taxonomy, reports of cases with abnormal oral sensations have accumulated over the years. There has been much discussion about treatment modalities [7,8,10,14-16] and potential biomarkers such as altered cerebral blood flow [9]. Descriptions of symptom characteristics and severities are subjective and limited by individual bias. Therefore, a standardized symptom assessment tool is necessary to facilitate scientific discussion among health care providers and researchers for identifying biomarkers and improving diagnosis and treatment modalities. Currently, there are no standard tools to measure and rate oral dysesthesia symptoms. In this study, we developed the Oral Dysesthesia Rating Scale (Oral DRS) and evaluated its validity as an assessment tool. We also evaluated its reliability by utilizing it in patients complaining of oral dysesthesia symptoms. Since patients often develop impairments in oral functions such as eating and speaking as well as in performance of daily activities, this new tool is designed to also assess these dysfunctions.

## Methods

### Development of Oral DRS

The Tokyo Medical and Dental University Oral DRS was developed based on literature review and extensive clinical experience. The literature included various case reports and a case–control study on oral dysesthesia [7,9,14-16], which provided information on the nature and diversity of symptoms as well as the influence on oral and daily functions. Oral DRS consists of a Symptom Severity Scale (SSS) [A], Functional Impairment Scale (FIS) [B], and Visual Analog Scale (VAS) [C] (Additional file 1). In the SSS [A], oral symptoms are classified into seven categories: feelings of foreign body [A1], exudation [A2], squeezing-pulling [A3], movement [A4], misalignment [A5], pain [A6], and spontaneous thermal sensation or tastes [A7]. The FIS [B] assesses the degree of impairment of eating [B1], articulation [B2], work [B3], and social activities [B4]. For each category, interviewers are required to identify symptoms or impairment and assess their severity in a semi-structured manner. For example, in the assessment of [A1], the interviewers start with leading questions “Do you feel something foreign is stuck in your mouth?” If the answer is yes, the interviewers rate its severity on a scale of 0–5 (0: none, 1: subtle or suspected, 2: mild, 3: moderate, 4: severe, 5: extremely severe); each severity level is explained in the scoring sheet to aid assessment. The VAS [C] consist of two scales that assess overall subjective severity of the symptoms [C1] and changes in severity of the symptoms [C2]. Each VAS is 100-mm long, where 0 mm is “No symptoms” and 100 mm is “Extremely severe” for [C1], and 0 mm is “Worse”, 50 mm is “no change”, and 100 mm is “Better” for [C2]. Patients are instructed to mark on the VAS sheet with a pen. The Oral DRS is available in Japanese and English with the respective instruction manuals on our website: (http://www.tmd.ac.jp/med/psyc/research/oral-drs.html). The validity and reliability of the Japanese version was assessed in this study.

### Content validity

Twelve reviewers assessed the content validity of Oral DRS to ensure that the included items are relevant to the purpose of the tool. Reviewers include eight psychiatrists and four dentists from our university hospital who had encountered oral dysesthesia in their practice but were not involved in the development of this tool. The reviewers were requested to evaluate each of the 11 items in the scales [A] and [B] on the score sheet as “1 = not relevant, 2 = somewhat relevant, 3 = quite relevant, or 4 = highly relevant”. Item content validity index (I-CVI) for each item was computed as the number of reviewers giving a rating of either 3 or 4, divided by the total number of reviewers [17]. An acceptable I-CVI is defined as 0.8 or above, while an average of 0.90 or above indicates excellent content validity [18]. Reviewers were also asked to choose one of the three statements regarding sufficiency of items for oral dysesthesia symptoms: 1. There are items that need to be added to the scales, 2. There are items that need to be omitted from the scales, 3. Items in the scales are necessary and sufficient. The reviewers were invited to provide feedback under a heading “any comments”.

### Inter-rater reliability

To evaluate the inter-rater reliability of Oral DRS, Oral DRS was utilized in 40 randomly selected patients who consulted the psychiatry clinic or psychosomatic dentistry clinic of the university hospital with complaints of oral dysesthesia symptoms. All the patients had been assessed by dentists and their symptoms were not regarded as organic or functional in origin. The raters consisted of psychiatrists or dentists who were instructed on how to perform semi-structured interviews using a written manual. Pairs of raters were formed, and each individual in the pair administered Oral DRS to the same patient separately on the same day. The first raters from the pair assessed patients using all three scales, while the second interviewers used [A] and [B] but omitted [C]. Cohen’s weighted kappa coefficient [19,20] was determined for each item in scales [A] and [B] in order to evaluate the inter-rater reliability for the tool. The R software (Vienna, Austria) version 2.13.0 with “psych” package was used for this analyses. Kappa agreement was defined a priori as excellent (kappa coefficient ≥ 0.8), good (≥0.6), moderate (≥0.4), fair (≥0.2), or poor (<0.2) [21]. This study was conducted with the approval of the Ethical Committee of Tokyo Medical and Dental University (No. 23–21, No. 356) and all participants gave written informed consent in accordance with the committee’s guidelines.

## Results

### Content validity

**Figure 1:**
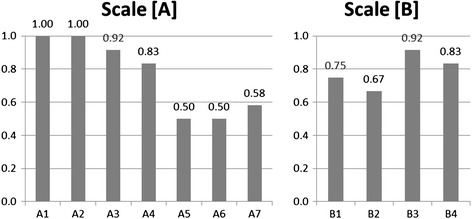
**Item content validity index for scales [A] and [B].** Item content validity indices for all items are shown. For the Symptom Severity Scale **[A]**, items A1, A2, A3, and A4 demonstrated acceptable content validity (≥0.8), while items A5, A6, and A7 did not reach acceptable values. For the Functional Impairment Scale **[B]**, while items B1 and B2 did not reach acceptable values, items B3 and B4 demonstrated acceptable content validity.

All reviewers completed the score sheet. I-CVI for each item of Oral DRS is shown in Figure 1. For the SSS [A], items A1 (foreign body), A2 (exudation), A3 (squeezing-pulling), and A4 (movement) demonstrated acceptable content validity (≥0.8) (1.00, 1.00, 0.92 and 0.83, respectively), while items A5 (misalignment), A6 (pain), and A7 (spontaneous thermal sensation or tastes) did not reach acceptable values (0.50, 0.50 and 0.58, respectively). For the FIS [B], items B3 (work) and B4 (social activities) demonstrated acceptable content validity (0.92 and 0.83, respectively), but items B1 (eating) and B2 (articulation) did not reach acceptable values (0.75 and 0.67, respectively). None of the reviewers chose “1 = not relevant” for any item in the scales. For the question regarding sufficiency of items, three reviewers chose “1. There are items that need to be added to the scales” and commented that “feeling of dry mouth”, “suicidal ideation”, and “depersonalization” should be considered for the assessment. All other reviewers chose “3. Items in the scales are necessary and sufficient” and added no comments.

### Inter-rater reliability

A total of 40 patients (8 men and 32 women) gave consent for the study and were assessed by Oral DRS. The demographics of the patients are shown in Table 1. The mean age of the subjects was 65.5 ± 10.0 (mean ± standard deviation) years. Duration of the illness at the time of the evaluation was 5.5 ± 3.9 years. The primary diagnoses were major depressive disorders for 13 patients, bipolar disorder for one patient, schizophrenia for one patient, and somatic-type delusional disorder for one patient. The remaining 24 patients were diagnosed with somatoform disorders.

**Table 1:** **Demographics**

Age (years)	65.5 ± 10.0^a^
Duration of illness (years)	5.5 ± 3.9^a^
Sex (male : female)	8 : 32
Primary diagnosis	
Somatoform disorders	24
Major depressive disorder	13
Bipolar disorder	1
Schizophrenia	1
Somatic-type delusional disorder	1
Medication^b^	
Antipsychotics	12
Antidepressants	18
Mood stabilizers	3
Anxiolitics and hypnotics	23

^a^average ± standard deviation. ^b^cumulative number of patients on each medication.

**Figure 2:**
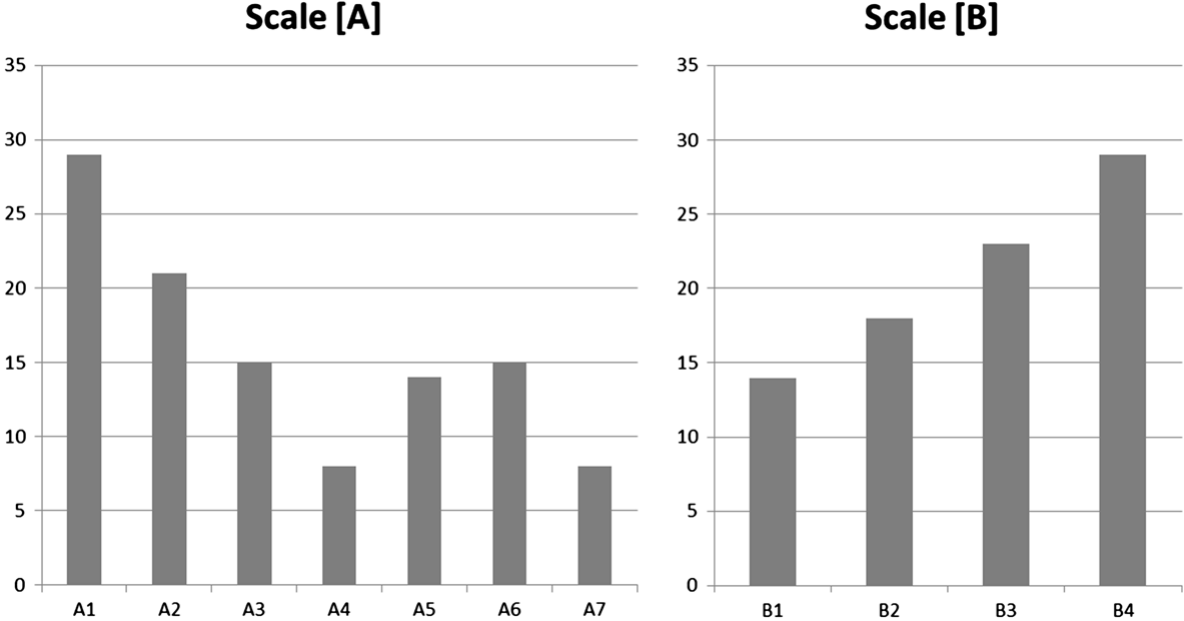
**Frequency distribution of the symptoms.** To overview the frequency distribution of the symptoms, the cumulative number of patients who were rated as “2: mild” or more for each item in scales **[A]** and **[B]** is shown. The distribution pattern tends to be similar to the pattern of I-CVI except for items A4 and A7 whose values are relatively low compared to those of I-CVI (Figure 1).

To overview the frequency distribution of the symptoms, the cumulative number of patients who were rated as “2: mild” or greater for each item in scales [A] and [B] are shown in Figure 2. The symptoms as in items A1 and A2 were relatively frequent, followed by items A3, A5 and A6, while the symptoms as in items A4 and A7 were relatively less frequent. The functional impairments as in items B4 was most frequent, followed by B3, B2 and B1 in descending order.

Cohen’s weighted kappa coefficients were calculated for each item in the [A] and [B] scales (Table 2). For the SSS [A], agreements between two raters, as defined by the kappa coefficient, were excellent (≥0.8) for items A1 (0.95), A2 (0.89), A3 (0.90), A4 (0.88), A5 (0.96) and A6 (0.83) and good (≥0.6) for item A7 (0.70). For the FIS [B], agreements between two raters were excellent for items B1 (0.87), B3 (0.93) and B4 (0.80) and good for item B2 (0.65).

**Table 2:** **Kappa coefficients**

A1. Foreign body	0.95
A2. Exudation	0.89
A3. Squeezing-pulling	0.90
A4. Movement	0.88
A5. Misalignment	0.96
A6. Pain	0.83
A7. Spontaneous thermal sensation or tastes	0.70
B1. Eating	0.87
B2. Articulation	0.65
B3. Work	0.93
B4. Social activities	0.80

Kappa coefficient were good (≥0.6) for items A7 and B2. For all other items, agreements were excellent (≥0.8).

## Discussion

We developed a novel assessment tool to quantitatively evaluate psychosomatic symptoms in oral regions and the consequent functional impairments. We also assessed its content validity and inter-rater reliability. Patients with oral dysesthesia experience unusual sensations and report their symptoms using various descriptions. We included multiple sensations in the SSS [A] after categorizing the nature of the reported symptoms.

Most reviewers felt items in the scales are necessary and sufficient. However, not all items in the scales demonstrated adequate content validity. It is possible that the reviewers feel symptoms or impairments are more relevant to the scales if they are encountered more frequently, and vice versa. Comparing Figure 1 (I-CVI) and Figure 2 (frequency distribution of the symptoms) helps to overview this tendency, where the first three items in the scale [A] and the last two items in the scale [B] are relatively frequent and regarded as more relevant. The item A4 (movement) demonstrated high I-CVI, while its frequency was relatively low. This discrepancy might be due to the bizarreness of the moving sensation as a symptom to the interviewers. That is, since moving sensation remind the interviews of delusional infestation [22], they may consider it is adequately relevant to the scale even though it is clinically rare. With regard to less frequent symptoms such as “salty taste in the mouth” [A7], some of the reviewers may have never encountered patients with these symptoms, yet these symptoms may be the only and most important concern of the patient. Therefore, we believe that the items with low I-CVI should be retained in the scales in order not to miss rare symptoms. Some reviewers commented that feeling of dry mouth, suicidal ideation, and depersonalization should be included in the scales. However, feeling of dry mouth was not included since it is a common medical complaint and not necessarily presented as dysesthesia. We may consider including it in a future revision if more users of the current tool demand it. One reviewer recommended the inclusion of suicidal ideation in the scale since oral dysesthesia is so uncomfortable in some patients that they feel suicidal. Therefore, the risk of suicide needs to be carefully evaluated for patients with oral dysesthesia, but other assessment tools such as the Hamilton Depression Rating Scale (HDRS) [23] can be simultaneously used with the Oral DRS for this purpose. It is important to note that a reviewer recommended including depersonalization in the scale, since some patients complain of this symptom along with abnormal oral sensations. Although cenesthopathy is considered to be associated with a state of depersonalization as discussed in a recent review article [2], we did not include it in the scale because it is not a direct representation of oral dysesthesia; instead, we recommend that it be noted in the margin of the scoring sheet.

Overall, the inter-rater reliability, as evaluated using Cohen’s kappa coefficients, was good, and we conclude that our tool is reliable for assessing symptoms of oral dysesthesia and the consequent functional impairments. The demographics of the patients included in this study provide an additional overview of typical characteristics of patients with oral dysesthesia as they were randomly recruited from our daily practice. Based on our clinical experience, a typical patient may be an elderly woman having this condition for relatively a long time with a history of anxiolytics, antidepressants, and/or antipsychotics use.

## Conclusion

Oral DRS is a reliable tool to quantitatively evaluate symptoms of oral dysesthesia and the consequent functional impairments. It is designed to cover various symptoms presented by patients by classifying them into seven main categories. In addition to being applied for clinical evaluation and follow-up, this tool can be utilized for clinical research on topics such as elucidating the association between severity of each symptom and findings of imaging studies.

## Additional file

Additional file 1:
**Oral Dysesthesia Rating Scale.** http://www.tmd.ac.jp/med/psyc/research/oral-drs.html.

## Abbreviations

Oral DRS: Oral Dysesthesia Rating Scale
DSM-IV: Diagnostic and statistical manual of mental disorders, fourth edition
ICD-10: ICD-10 Classification of mental and behavioural disorders
SSS: Symptom severity scale
FIS: Functional impairment scale
VAS: Visual analog scale
I-CVI: Item content validity index
HDRS: Hamilton depression rating scale
