# Supplementary material for: Oral Dysesthesia Rating Scale: a tool for assessing psychosomatic symptoms in oral regions
Source: BMC Psychiatry. 2014 Dec 21;14:1696. doi: 10.1186/s12888-014-0359-8 (PMC4300025; doi:10.1186/s12888-014-0359-8)
Supplement: Additional file 1: — Oral Dysesthesia Rating Scale. http://www.tmd.ac.jp/med/psyc/research/oral-drs.html. [file 12888_2014_359_MOESM1_ESM.pdf]

Name: ID: Date: Rater:

Oral DRS

Tokyo Medical and Dental University **O**ral **D**ysesthesia **R**ating **S**cale

A. Symptom Severity Scale (SSS)		0: none	1: very mild	2: mild	3: moderate	4: severe	5: very severe	score
**A1. Foreign body**	Foreign body sensation. Description: sticky, sandy, nail-like, or wire-like objects stuck between the teeth or in the mouth, attachment of foreign object, etc.	None	Suspected or borderline. Very mild symptoms.	Infrequent and mild symptoms.	Frequent, moderate but tolerable symptoms. Maybe alleviated by wiping, brushing, etc.	Very frequent and relatively intolerable symptoms.	Constant and extremely intolerable symptoms, accompanied by agitation or incessant complaining.	
**A2. Exudation**	Sensation of exudation, welling out, or gushing of saliva, bubbles, mucus, sand, etc.	None	Suspected or borderline. Very mild symptoms.	Infrequent and mild symptoms.	Frequent, moderate but tolerable symptoms. Maybe alleviated by use of mouthpiece, etc.	Very frequent and relatively intolerable symptoms.	Constant and extremely intolerable symptoms, accompanied by feelings of physical impairment such as inability to close the mouth.	
**A3. Squeezing -pulling**	Sensation of squeezing or pulling of the teeth, lips, etc.	None	Suspected or borderline. Very mild symptoms.	Infrequent and mild symptoms.	Frequent, moderate but tolerable symptoms.	Very frequent and relatively intolerable symptoms, accompanied by pain.	Constant and extremely intolerable symptoms. May be accompanied by face distortion or use of hands or instruments to alleviate symptoms.	
**A4. Movement**	Sensations of involuntary movement of the teeth, lips, or jaw; insect infestation; or circulating fluid in the gums.	None	Suspected or borderline. Very mild symptoms.	Infrequent and mild symptoms.	Frequent, moderate but tolerable symptoms.	Very frequent and relatively intolerable symptoms.	Constant and extremely intolerable symptoms, accompanied by agitation or incessant complaining.	
**A5. Misalignment**	Sensation of misaligned jaws or teeth. Description: shifted teeth or jaws, enlarged teeth, positional discomfort, and physical misalignment.	None	Suspected or borderline. Very mild symptoms.	Infrequent and mild symptoms.	Frequent, moderate but tolerable symptoms.	Very frequent and relatively intolerable symptoms.	Constant and extremely intolerable symptoms, accompanied by agitation or incessant complaining.	
**A6. Pain**	Sensation of pain. Description: Pricking, searing, burning, general pain, etc.	None	Suspected or borderline. Very mild symptoms.	Infrequent and mild symptoms.	Frequent, moderate but tolerable symptoms.	Very frequent and relatively intolerable symptoms.	Constant and extremely intolerable symptoms, accompanied by agitation or incessant complaining.	
**A7. Spontaneous thermal sensation or tastes**	Spontaneous thermal sensations or tastes. Description: hot, warm, cold, cool, sweet, bitter, sour, salty, etc. Does not include simple thermal or gustatory anesthesia.	None	Suspected or borderline. Very mild symptoms.	Infrequent and mild symptoms.	Frequent, moderate but tolerable symptoms.	Very frequent and relatively intolerable symptoms.	Constant and extremely intolerable symptoms, accompanied by agitation or incessant complaining.	
					Intermediate scores such as 1.5 and 2.5 are allowed.		**A. SSS Total**	

Download the latest version at: http://www.tmd.ac.jp/med/psyc/research/oral-drs.html

Created 2011.6.29 Last minor modification 2014.11.26

Name: ID: Date: Rater:

B. Functional Impairment Scale (FIS)		0: none	1: very mild	2: mild	3: moderate	4: severe	5: very severe	Score
**B1. Eating**	Disturbance of eating function and altered dietary preference.	None	Feels some difficulty but eats normally.	Feels mild difficulty. Somewhat decreased ingestion. Altered dietary preference, e.g., avoids sticky foods.	Feels moderate difficulty. Significantly decreased ingestion. Some decrease in body weight.	Feels severe difficulty. Significantly decreased body weight.	Impossible to eat. Needs gastrostomy or parenteral nutrition.	
**B2. Articulation**	Disturbance of speech or articulation.	None	Feels some difficulty in speech or articulation, but sounds normal to others.	Feels mild difficulty in speech or articulation. Sounds somewhat unnatural to others.	Feels moderate difficulty in speech or articulation. Sounds awkward and indistinct.	Feels severe difficulty in speech or articulation. Prominently awkward and indistinct.	Impossible to speak or articulate. Gives up spontaneous speech.	
**B3. Work**	Disturbance of social function/work. Examples: housework for a homemaker and schoolwork for a student. Not limited to disturbance due to oral dysesthesia.	None<br><br>Close to 100%	Feels some difficulty but performs normally.<br><br>Greater than 90%	Feels mild difficulty with mild decline in efficiency.<br><br>"About 80%"<br><br>89–70%	Feels moderate difficulty. Significant decline in efficiency.<br><br>"About 50%"<br><br>69–30%	Feels severe difficulty. Prominent decline in efficiency. Hard to continue.<br>"Less than 30%"<br><br>>29%	No potential to perform.<br><br>Almost 0%	
**B4. Social Activities**	Disturbance of interpersonal (e.g., meeting friends, going out) or recreational (e.g., reading, exercise) activities. Not limited to disturbance due to oral dysesthesia.	None<br><br>Close to 100%	Feels some difficulty but performs normally.<br><br>Greater than 90%	Feels mild difficulty. Mild decline in activity.<br><br>"About 80%"<br><br>89–70%	Feels moderate difficulty. Significant decline in activity.<br><br>"About 50%"<br><br>69–30%	Feels severe difficulty. Prominent decline in activity. Hard to continue.<br>"Less than 30%"<br><br>>29%	No potential to perform.<br><br>Almost 0%	
					Intermediate scores such as 1.5 and 2.5 are allowed.		**B. FIS Total**	

Name: ID: Date: Rater:

C. Visual Analog Scale (VAS)

C1. Considering your experiences, how severe are your present symptoms?

| No symptoms | |————————————————————| | Extremely severe |

_______ mm

C2. Compared to the baseline (before treatment), how much have your symptoms improved or worsened?

| Worse | |————————————————————| | Better |

_______ mm

Download the latest version at: http://www.tmd.ac.jp/med/psyc/research/oral-drs.html

Created 2011.6.29 Last minor modification 2014.11.26

Name: ID: Date: Rater:

(Note)

Oral Dysesthesia Rating Scale: A tool for assessing psychosomatic symptoms in oral regions

Akihito Uezato[*1], Akira Toyofuku, Yojiro Umezaki, Akira Toriihara, Makoto Tomita, Naoki Yamamoto, Akeo Kurumaji, Toru Nishikawa[*2]

[*1] Contacting author [*2] Corresponding author

Department of Psychiatry and Behavioral Sciences

Tokyo Medical and Dental University Graduate School

1-5-45, Yushima, Bunkyo-ku, Tokyo 113-8519, Japan

E-mail: uezapsyc@tmd.ac.jp

Download the latest version at: http://www.tmd.ac.jp/med/psyc/research/oral-drs.html *Created 2011.6.29 Last minor modification 2014.11.26*
